# Ovarian Mesonephric-like Adenocarcinoma: Its Prevalence in a Japanese High-Volume Cancer Center and a Literature Review on Therapeutic Targets

**DOI:** 10.3390/curroncol31090378

**Published:** 2024-08-30

**Authors:** Ayako Ogawa, Hiroshi Yoshida, Saria Kawano, Nao Kikkawa, Mayumi Kobayashi-Kato, Yasuhito Tanase, Masaya Uno, Mitsuya Ishikawa

**Affiliations:** 1 Department of Gynecology, National Cancer Center Hospital, Tokyo 104-0045, Japanmaykobay@ncc.go.jp (M.K.-K.); yatanase@ncc.go.jp (Y.T.);; 2 Department of Diagnostic Pathology, National Cancer Center Hospital, 5-1-1 Tsukiji, Chuo-ku, Tokyo 104-0045, Japan; 3 Department of Diagnostic Radiology, National Cancer Center Hospital, Tokyo 104-0045, Japan

**Keywords:** homologous recombination repair, immunohistochemistry, mesonephric-like adenocarcinoma, ovary, therapeutic target

## Abstract

Background: Ovarian mesonephric-like adenocarcinoma (MLA) is a newly described histological type known for its aggressive behavior. This study aims to determine the frequency of ovarian MLA, review the existing literature, and elucidate its clinicopathological characteristics, including the potential therapeutic targets. Methods: We retrospectively reviewed the pathological diagnoses of 501 primary ovarian cancer surgical cases at our institution from 2010 to 2023. MLAs exhibiting typical morphological and immunohistochemical features were included. The frequency and clinicopathological characteristics of these cases were summarized. Additionally, we conducted a literature search using PubMed to collect and summarize previously reported cases of ovarian MLAs. Results: Among the 501 primary ovarian cancer cases, we identified 3 cases (0.6%) of MLA. The patients were 52–76 years old, and the initial FIGO stages were IC1 (two cases) and IIIB (one case). All the cases exhibited HRP, pMMR, PD-L1 negativity (CPS < 1), and low HER2 expression. Two cases experienced metastatic recurrence. A literature review identified 97 cases of MLA. The MLAs frequently exhibited *KRAS* mutations (90%, 38/42), with a recurrence rate of 39% (26/67). Conclusion: MLAs accounted for 0.6% of malignant ovarian tumors at our institution, all of which were advanced or recurrent cases. These cases showed HRP, pMMR, and PD-L1 negativity, indicating a lack of current therapeutic targets. The literature also reported a high incidence of advanced and recurrent cases, highlighting the need for accurate diagnosis and the development of new treatments. The frequent *KRAS* mutations suggest a potential therapeutic target for recurrent or metastatic MLA.

## 1. Introduction

Mesonephric-like adenocarcinoma (MLA) of the ovary is a newly classified histological subtype added to the 2020 World Health Organization (WHO) classification of female genital tumors. This tumor shares histopathological characteristics with mesonephric adenocarcinoma of the cervix, which originates from mesonephric duct remnants [1]. Most MLAs are reportedly associated with endometriosis, suggesting that it is one of the tumors related to endometriosis [2,3].

Although MLA has similarities to low-grade endometrioid carcinoma, distinguishing features have been reported in terms of its morphological and immunohistochemical characteristics. Morphologically, the tumor cells, which are cuboidal to columnar, proliferate in various architectural patterns, including tubular, papillary, and solid structures, often containing eosinophilic material within the glandular lumina [4]. Immunohistochemically, MLA is characterized by positive staining for TTF-1, GATA3, calretinin, and CD10 [1] and negative staining for estrogen receptor (ER), progesterone receptor (PR), and WT-1. Recognizing this histological type is crucial because, unlike low-grade endometrioid carcinoma, MLAs are reportedly associated with a poor prognosis and show a tendency for advanced stages and early recurrence [5].

Ovarian MLA is a rare tumor, expected to constitute less than 1% of ovarian tumors, similar to MLA in endometrial carcinoma, but its exact prevalence remains undetermined. Furthermore, reports indicate frequent cases of advanced stages and early recurrences, highlighting the need for therapeutic development. However, knowledge regarding molecular abnormalities that could serve as therapeutic targets in MLA is still insufficient.

This study aims to determine the prevalence of ovarian MLA and, by incorporating findings from the existing literature, elucidate its clinicopathological characteristics, focusing on potential therapeutic targets.

## 2. Materials and Methods

### 2.1. Patient Selection and Tissue Section Preparation

The present study was conducted in accordance with the Declaration of Helsinki and Good Clinical Practice and with the approval of the Institutional Review Board of the National Cancer Center, Tokyo, Japan (2020-111, 2010-077). Data from January 2010 to June 2023 were used, collecting a total of 683 consecutive patients with ovarian tumors. After the exclusion of 182 cases of borderline tumors, malignant germ cell tumors, or metastatic tumors, 501 cases of primary ovarian cancer were included in this study.

### 2.2. Pathological Diagnosis

At least two board-certified pathologists initially reviewed all the cases, and the pathological diagnoses in this study were subsequently confirmed by a gynecological pathologist (H.Y.) according to the 2020 WHO classification. Pathological diagnoses of the MLA were confirmed based on the previously reported morphological features and immunohistochemical findings [4]. MLAs should exhibit a morphological similarity to mesonephric adenocarcinoma of the uterine cervix. Different architectural patterns were observed, such as tubular, glandular (pseudo-endometrioid), papillary, cribriform, slit-like, retiform, glomeruloid, and solid, in various combinations. Intraluminal eosinophilic colloid-like material was frequently observed. Metaplastic changes such as squamous, ciliated, and mucinous differentiation are generally absent. Tumor cells are cuboidal or columnar cells with mild or moderately atypical angulated clear vesicular nuclei, often overlapping. The cytoplasm is usually scant to moderate, and mitotic activity is usually conspicuous. Immunohistochemically, the tumor cells show diffuse positivity of PAX8 and focal positivity of TTF-1, GATA3, CD10 (apical/luminal), and calretinin, as well as negativity of ER, PR, and WT-1. A p53 wild-type staining pattern and retained MMR proteins (MSH6 and PMS2) are also observed. One case of ovarian MLA was reported as a single case report [6]. The criteria for the diagnosis of MLA were summarized in Supplementary Table S1, and the primary antibodies used for immunohistochemistry for IHC were listed in Supplementary Table S2.

### 2.3. Literature Review

The literature search was performed using publications indexed in PubMed (http://www.ncbi.nlm.nih.gov/pubmed) accessed on 29 December 2023 from February 1964 to December 2023. The following search terms were used in the PubMed database: “mesonephric-like adenocarcinoma AND ovary”, or “ovarian mesonephric-like adenocarcinoma”. The reference lists of the included articles were manually checked for any undetected cases.

### 2.4. Statistical Analysis

Baseline characteristics were presented as frequencies and proportions for categorical variables. Continuous variables were presented as medians with ranges. All statistical analyses and graphic presentations were performed using SPSS (version 13.0J; SPSS Inc., Chicago, IL, USA).

## 3. Results

### 3.1. Clinical Characteristics

Of 501 cases of primary ovarian cancer, MLA was observed in 3 cases (0.6%). Each histological type and its prevalence are presented in Table 1.

The clinical features of these three cases are summarized in Table 2.

The patients’ ages ranged from 52 to 76 years, and all cases were postmenopausal. None of the cases were obese, and the initial symptoms included pelvic pain or the identification of an ovarian tumor due to a pelvic mass. All cases showed elevated CA125 levels, and CA19-9 was elevated in the two cases where it was measured. The maximum tumor diameter on imaging ranged from 13 to 16.5 cm. According to the International Federation of Gynecology and Obstetrics (FIGO, 2008), the initial stages were IC1 in two cases and IIIB in one case. Primary debulking surgery was performed in the two IC1 cases. The IIIB case underwent four courses of dose-dense Paclitaxel Carboplatin (ddTC) therapy as neoadjuvant chemotherapy, followed by interval debulking surgery and two additional courses of ddTC therapy. The initial pathological diagnosis was grade 1 endometrioid carcinoma in one case and mesonephric-like adenocarcinoma (MLA) in two cases. Recurrence was observed postoperatively in both the IC1 cases at 1 and 16 months, respectively. The recurrence sites were the lung in one case and the lung and liver in the other, followed by chemotherapy. The postoperative follow-up period ranged from 8 to 42 months, with outcomes of dead of disease in one case, alive with disease in one case, and no evidence of disease in one case.

### 3.2. Pathological Findings

**Figure 1:**
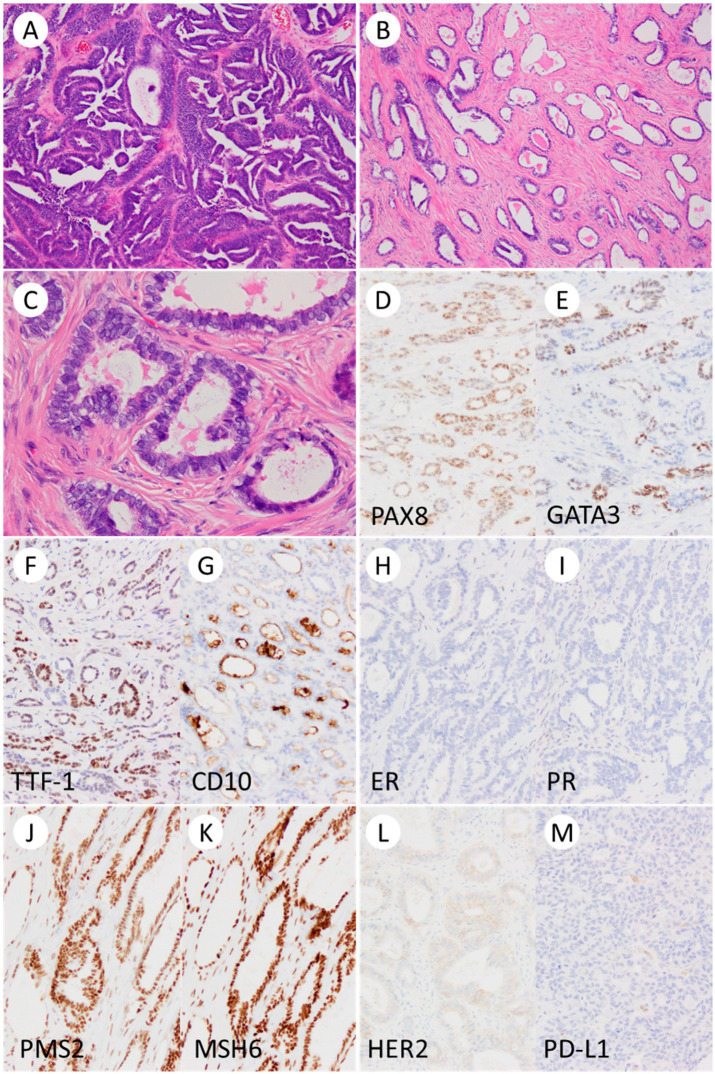
Morphological and immunohistochemical findings of a mesonephric-like adenocarcinoma of the ovary. The tumor shows various histological patterns, including glandular and papillary patterns ((**A**), ×100) and tubular structures containing eosinophilic material in the hyalinized stroma ((**B**), ×100). The tumor cells are cuboidal to cylindrical with enlarged nuclei ((**C**), ×100). Immunohistochemically, the tumor cells are positive for PAX8, GATA3 (focal), TTF1 (focal), and CD10 (luminal) but negative for estrogen receptor and progesterone receptor ((**D**–**I**), ×200). In addition, the tumor cells show intact expression of PMS2 and MSH6 ((**J**,**K**), ×200), are weakly positive for HER2 (focal; score 1+, ×200) (**L**), and are PD-L1 negative (combined positive score < 1) ((**M**), ×200).

The pathological features of the three cases are summarized in Table 3 and representative histology is shown in Figure 1.

Figure 1 shows the morphological and immunohistochemical findings of a mesonephric-like adenocarcinoma of the ovary.

Macroscopically, all tumors had both solid and cystic components. Histologically, the tumors showed a mixed pattern of glandular and papillary structures, with eosinophilic colloid-like material inside the glands. The tumor cells ranged from cuboidal to columnar, with no nuclear pleomorphism, although some cells exhibited ground glass nuclei and nuclear overlap. Endometriosis was observed in the background of all cases. Immunohistochemically, all cases were positive for GATA3, TTF-1, CD10, and calretinin but negative for the estrogen and progesterone receptors and WT-1. All cases exhibited a wildtype p53 pattern and a retained expression of MMR proteins. Additionally, programmed death-ligand 1 (PD-L1) was negative (combined positive score, CPS < 1). The HER2 score for all tumors was 1+. The commercially available companion HRD test (MyChoice^®^) provided by Myriad Genetics was performed using the ovarian tumor tissue, revealing that homologous recombination repair was proficient in all the cases (genomic instability score was less than five in all the three cases, and neither the BRCA1 nor the BRCA2 pathogenic variant was present).

### 3.3. Literature Review and Summary of Previously Reported Cases

A literature search on PubMed identified 97 cases of ovarian MLA with confirmed pathological diagnoses (Table 4 and Table 5) [1,5,7,8,9,10,11,12,13,14,15,16,17,18,19,20,21,22,23,24,25,26,27,28,29,30,31,32].

The ages ranged from 29 to 84 years. According to FIGO, the initial stages were I in 33 cases, II in 12 cases, III in 15 cases, and IV in 7 cases. A significant proportion (84%, 21/25 cases) were associated with endometriosis. Recurrence was reported in 39% (26/67 cases). A total of 3 patients died among the 23 patients with available follow-up data. Notably, *KRAS* mutations were observed in 90% (38/42 cases) of the patients. There has been minimal investigation into therapeutic target molecules, and no cases of HRD or MMRd have been identified to date. PD-L1 positivity has also not been reported.

## 4. Discussion

We identified 3 cases (0.6%) of MLA out of the 501 primary ovarian cancer cases based on morphological and immunohistochemical staining results. Two cases showed metastatic recurrence, for which chemotherapy was initiated. All cases were HRP, pMMR, negative for PD-L1 (CPS < 1) and had a HER2 score 1+. The literature review identified 97 previously reported cases, confirming that advanced cases are common and have a high recurrence rate (39%). The molecular features associated with therapeutic targets in these cases were similar to our three cases, but a high frequency of *KRAS* mutations was observed, suggesting potential targets for therapy.

Ovarian MLA is predicted to be a rare tumor among ovarian neoplasms, similar to MLA in endometrial cancer, but there have been no reports on the frequency of ovarian MLA. In contrast, endometrial MLA has been reported to occur at a frequency of approximately 0.7% of all endometrial cancers [8,33]. In a combined morphological and molecular analysis of 570 endometrial carcinomas, only 4 cases (0.7%) were diagnosed as MLA [33]. Pors et al. (2018) also reported a similar frequency based on an analysis of 585 endometrial carcinomas between 1986 and 2017 [8]. Our investigation revealed that ovarian MLA, similar to endometrial MLA, occurs at a frequency of 0.6% (3/501, 95% CI 0.12~1.8%) in a Japanese cohort.

Additionally, one of our three cases had been diagnosed as grade 1 endometrioid carcinoma. Before the establishment of the MLA concept, some MLA cases might have been diagnosed as low-grade endometrioid carcinoma. In cases of low-grade endometrioid carcinoma that show unusual clinical courses, such as early recurrence or distant metastasis, reconsideration of the diagnosis to MLA might be warranted.

Our three cases, along with the 97 cases identified through the literature review, suggest that MLA has more aggressive clinicopathological features compared to low-grade endometrioid carcinoma. Clinically notable, our literature review results show that approximately 12% of low-grade endometrioid carcinomas are reportedly stage III/IV [34], and 33% of MLAs are at stage III/IV. Additionally, while the recurrence rate of low-grade endometrioid carcinoma is around 9% [35], 39% of ovarian MLAs are reported to recur based on our literature review results. Furthermore, distant metastasis occurs in 33–56% of MLA cases [5,15]. Among our three cases, one was detected at an advanced stage, and two experienced distant metastatic recurrence. Histopathologically, compared to low-grade endometrioid carcinoma, MLAs typically have fewer solid components and more prominent gland formation. They lack the metaplastic changes such as squamous and mucinous metaplasia which are often seen in endometrioid carcinoma and instead exhibit cellular morphology that is more cuboidal than tall columnar. If immunostaining shows negative hormone receptor status, additional stains such as GATA3, TTF-1, calretinin, and CD10 can aid in diagnosing an MLA [4]. Consistently, in the molecular classification of endometrial carcinoma (POLEmut, dMMR, p53abn, and NSMP), MLAs display an NSMP profile characterized by wildtype POLE, pMMR, and wildtype TP53, which can further support the diagnosis.

Mismatch repair deficiency (dMMR) and PD-L1 expression in tumor cells or the surrounding immune cells are known predictors of the efficacy of immune checkpoint inhibitors across various tumors [36]. Multiple studies have reported that ovarian MLA exhibits proficient mismatch repair (pMMR) [15,20]. Our literature review also found that all cases with an evaluated MMR status were pMMR (100%, 35/35 cases). In contrast, approximately 8–19% of ovarian endometrioid carcinomas show dMMR [37], suggesting that pMMR might be a distinguishing feature of MLA from endometrioid carcinoma. However, the lack of increased tumor neoantigen production and immunogenicity in pMMR tumors indicates a lower likelihood of response to immune checkpoint inhibitors. Regarding PD-L1 expression in MLA, only one case has been reported as negative [12], and similarly, in our cases, PD-L1 expression was minimal in both the tumor and the surrounding immune cells. Although further studies with larger sample sizes are needed, the current evidence suggests that immune checkpoint inhibitors might be ineffective for advanced or recurrent MLA.

In our study, we reported an HR status, for the first time, in multiple MLA cases. Previously, only one case had been reported as HRP [17], and our three cases were also HRP. While approximately half of the high-grade serous carcinoma (HGSC) cases show homologous recombination deficiency (HRD) (Cancer Discovery, 2015), endometriosis-associated ovarian cancers are reported to have lower HRD rates compared to HGSC [38]. This suggests that ovarian MLA, like other endometriosis-associated ovarian cancers, may predominantly exhibit HRP. Since HRD is a predictive factor for the efficacy of PARP inhibitors, the effectiveness of PARP inhibitors for advanced or recurrent MLA may be limited, given the HRP status.

On the other hand, *KRAS* mutations might be a promising therapeutic target in MLAs. Although we did not test for *KRAS* mutations in our cases, our literature review found that *KRAS* mutations were identified in 90% (38/42 cases) of the tested cases, including specific mutations like G12V (42%, 16/38 cases), G12D (39%, 15/38 cases), and G12A (8%, 3/38 cases). Recent developments in targeted therapies for *KRAS* mutations in the lung and other major cancers suggest potential cross-organ applicability. For example, the *KRAS* inhibitor sotorasib showed a 37% objective response rate in advanced or recurrent non-small cell lung cancer with *KRAS* p.G12C mutation [39]. Although most *KRAS* mutations in MLA are not p.G12C, ongoing research targeting other *KRAS* mutations might eventually benefit MLA treatment.

Additionally, although our analysis is limited to three cases, all exhibited weak HER2 protein expression (Score 1+). Recent studies have shown the clinical efficacy of HER2-ADC in low-HER2/HER2-expressing cancers, such as breast cancer [40], indicating that recurrent or metastatic MLA might also be a candidate for such therapies. Beyond HER2, the development of various ADCs is ongoing, and identifying the target molecules in MLA is a crucial future research direction.

This study is a retrospective analysis conducted at a single high-volume cancer center, and the disease frequency distribution may differ from that of general hospitals. Therefore, this selection bias may influence the frequency of the MLAs identified in our study. Furthermore, our institution had only three cases to investigate therapeutic target molecules. Hence, the findings need to be validated through multicenter studies with larger cohorts of MLA cases. Additionally, this study did not investigate *KRAS* gene mutations. Although *KRAS* mutation testing is not mandatory for diagnosing MLA [4], identifying *KRAS* mutations could provide additional support.

In summary, an analysis of 501 primary ovarian cancer cases revealed that the frequency of ovarian MLA is 0.6%. Ovarian MLA exhibits HRP, pMMR, PD-L1 negativity, and low-HER2 expression, suggesting that the effectiveness of PARP inhibitors and immune checkpoint inhibitors may be limited. However, the high prevalence of *KRAS* mutations indicates that *KRAS* mutation could be a potential therapeutic target for recurrent or metastatic MLA. Given the high incidence of advanced, recurrent, and metastatic cases in MLA, it is essential to validate these therapeutic target findings in larger cohorts.

## Figures and Tables

**Table 1:** Histological type of 501 primary ovarian cancers.

Histological Type	*n* (%)
	Total *n* = 501
High-grade serous carcinoma	239 (46.3)
Clear cell carcinoma	116 (22.5)
Endometrioid carcinoma	57 (11.0)
Mucinous carcinoma	30 (5.8)
Adenocarcinoma, unclassifiable *	17 (3.3)
Low-grade serous carcinoma	16 (3.1)
Mixed cell carcinoma	11 (2.1)
Carcinosarcoma	8 (1.6)
**Mesonephric-like adenocarcinoma**	**3 (0.6)**
Undifferentiated carcinoma	2 (0.4)
Malignant Brenner tumor	1 (0.2)
Squamous cell carcinoma	1 (0.2)

* Small amounts of residual adenocarcinoma after chemotherapy.

**Table 2:** Clinical characteristics of 3 cases of ovarian mesonephric-like adenocarcinoma.

	Case 1	Case 2	Case 3
Age [year]	76	52	71
Obstetric history	G3P3	G0P0	G0P0
Menopause [year]	58	50	51
Previous medical history	rheumatoid arthritis	leiomyoma, lt shoulder fracture	breast cancer, dyslipidemia
Body mass index [kg/m^2^]	18	22.9	22.5
Symptom	pelvic pain, abdominal distension	adnexal mass	pelvic pain, pelvic mass
Tumor marker	CA19-9: 290 U/mL, CA125: 434 U/mL	CA19-9: 135 U/mL, CA125: 64 U/mL	CA125: 199 U/mL
Radiological diagnosis	rt ovarian cancer	lt ovarian cancer (s/o EM, CCC)	ovarian cancer
Clinical stage (FIGO 2008)	cT1N0M0	cT1N0M0	cT3bN0M0
Surgical procedure	PDS, TAH+BSO+OMT+PLNB	PDS, TAH+BSO+OMT+PLNB	IDS, TAH+BSO+OMT+PLNB+LAR
Pathological stage	pT1c1N0M0	pT1c1N0M0	ypT2N0M0
Tumor size [cm]	15.5	13	16.5
Recurrence [month]	Yes, 16 month	Yes, 1 month	No
Metastatic site	liver, lung	liver	-
Follow-up time [month]	42	9	8
Prognosis	DOD	AWD	NED

Abbreviations: PDS, primary debulking surgery; TAH, total abdominal hysterectomy; BSO, bilateral salpingo-oophorectomy; OMT, omentectomy; PLNB, pelvic lymph node biopsy; LAR, low anterior resection of rectum; DOD, dead of disease; AWD, alive with disease; NED, no evidence of disease.

**Table 3:** Pathological features of 3 cases of ovarian mesonephric-like adenocarcinoma.

	Case 1	Case 2	Case 3
Laterality	bilateral	left	bilateral
Tumor size [cm]	rt. 15 × 11.5 × 9; lt. 4 × 3 × 1.5	16 × 10 × 4	rt. 3.5 × 2.5 × 1; lt. 11.5 × 7 × 2
Macroscopic type	solid and cystic	solid and cystic	solid and cystic
Glandular and papillary pattern	+	+	+
Intraluminal eosinophilic secretion	+	+	+
Spindled tumor cells	+	+	+
Sex cord-like pattern	+	-	+
Hyalinized/Fibrous stroma	+	+	+
Tumor infiltrating lymphocytes	a few	a few	a few
Glassy nucleus	+	+	+
Mitotic counts	up to 14/10HPFs	up to 10/10HPFs	up to 11/10HPFs
Metaplasia	no	no	no
Endometriosis	+	+	+
Endometrium	atrophic	atrophic	atrophic
Adenomyosis/Leiomyoma	−/+	+/+	+/+
Immunohistochemistry			
ER/PR/WT-1	−/−/−	−/−/−	−/−/−
GATA3/TTF-1 positivity	focal/focal	focal/focal	diffuse/focal
CD10/Calretinin positivity	focal/focal	focal/focal	focal/rare
p53	wild-type pattern	wild-type pattern	wild-type pattern
MMR	pMMR	pMMR	pMMR
PD-L1 (22C3)	CPS < 1	CPS < 1	CPS < 1
HER2	score 1+	score 1+	score 1+
HR (myChoice^®^)	HRP (GIS = 4)	HRP (GIS = 1)	HRP (GIS = 2)

Abbreviations: pMMR, mismatch repair proficient; CPS, combined positive score; HRP, homologous recombination proficient; GIS, genomic instability score.

**Table 4:** Clinicopathological summary of previously reported cases of mesonephric-like adenocarcinoma of the ovary.

Author/Year	*n*	Age	Laterality	Size (cm)	Surgical Treatment	FIGO Stage	Recurrence	Survival	Follow-Up Time (Month)
McFarland/2016 [1]; Mirkovic/2018 [7]	5	42–62 (4); N/A (1)	B (2); L (2); N/A (1)	4–32	TH + BSO (2); BSO (1); N/A (2)	IA (1); IC (1); IIB (1); IIIC (1); N/A (1)	Yes (1); No (4)	Alive (5)	7–37 (4); N/A (1)
Pors/2018 [8]	1	67	N/A	N/A	N/A	IC	N/A	N/A	N/A
Chapel/2018 [9]	1	80	R	10.6	TH+BSO+OMT+P	IIIC	No	Alive	3
McCluggage/2020 [10]	5	50–77	R (1); L (3); N/A (1)	6 (1); N/A (4)	TH+BSO+PLND+OMT+P (1); N/A (4)	IIIA (1); NA (4)	N/A	N/A	N/A
Dundr/2020 [11]	1	61	L	3.5	TH+BSO+OMT+P+A	IVB	No	Alive	N/A
Seay/2020 [12]	1	67	R	11	TH+RSO+PLND+OMT	IA	Yes, abdominopelvic	Alive	18
Chen/2020 [13]	1	29	R	10	TH+BSO+PLND+PALND+OMT	IC2	No	Alive	13
Qazi/2020 [14]	1	51	N/A	18	N/A	N/A	N/A	N/A	N/A
Pors/2021 [5]	25	36–81	N/A	N/A	N/A	I (11); II–IV (7); N/A (7)	Yes (10); No (14); N/A (1)	5-yr OS 71% (23)	101 (mean)
da Silva/2021 [15]	15	36–76	B (1); R (2); N/A (12)	3.5–18.5 (12); N/A (3)	N/A	IA (2); IC (3); IIB (2); IIIA (1); IIIC (2); IV (3); NA (2)	Yes (10: abdominopelvic, 6;distant metastasis, 4)	N/A	N/A
Kim/2021 [16]	1	47	L	4.4	PLND+PALND+OMT+P	IIIC	No	Alive	11
Karpathiou/2021 [17]	1	74	L	19	TH+OMT+P+LND	IIIB	No	Alive	6
Ujita/2021 [18]	1	84	L	7	TH+BSO+pOMT	IC3	No	Alive	4
Deolet/2022 [19]	4	33–75	R (1); L (2); N/A (1)	7–15 (3); N/A (1)	LSO (1); TH+BSO+OMT (1); BSO (1); cyctectomy (1)	IA (1); IC (1); IIIC (1); IVB (1)	Yes, abdominopelvic (1); No (3)	Alive (4)	8–46
Koh/2022 [20]	5	42–61	R (2); L (3)	4.7–11.0	TH+BSO+PLND+PALND+P+OMT (1); TH+BSO+PLND+P+OMT (1); BSO+PLNb+P+OMT (1); TH+BSO+PLND+PALND+Pb+OMT (1); TH+BSO+P (1)	IA (1); IC (3); IIB (1)	Yes, distant metastasis (1); No (3); N/A (1)	Dead (1); Alive (3); N/A (1)	11–53 (4); N/A (1)
Ishida/2022 [21]	1	69	B	3.2, 2.0	TH+BSO	IIB	Yes, lung	N/A	N/A
Arslanian/2022 [22]	2	66–67	R (1); L (1)	8, 18	TH+BSO+infracolic omentectomy+rightPALND(1); TH+BSO+PLND+OMT (1)	IC (1); IIIA1 (1)	-	Dead (1); Alive (1)	15–32
Nilforoushan/2022 [23]	2	55–58	L (2)	12, 13	TH+BSO+OMT (1); TH+LSO+OMT+LND+pelvic staging biopsy (1)	N/A	N/A	N/A	N/A
Mirkovic/2023 [24]	2	61–62	R (2)	9, 27	TH+BSO+OMT+LAR (1); BSO+OMT+rectosigmoid and posterior vaginal ressection (1)	IIB (2)	N/A	Alive (2)	12, 6
Xu/2023 [25]	1	78	R	4.3	TH+BSO+OMT+PLND	IC2	Yes, pelvic	N/A	60
Nilforoushan/2023 [26]	1	70	B	6.2, 2.9	TH+BSO+OMT	IVB	N/A	N/A	N/A
Stolnicu/2023 [27]	1	63	L	12	TH+BSO	IC	N/A	N/A	N/A
Kommoss/2023 [28]	14	50–83	N/A	N/A	N/A	N/A	N/A	N/A	N/A
Zhao/2023 [29]	1	58	B	10, 24	TH+BSO	IC, IIA	N/A	N/A	N/A
Chang/2023 [30]	2	51–57	R (2)	9.6, 5.7	TH+BSO+OMT+rt PLND (1); RATH+BSO+PLND (1)	IIIA1, IA1	N/A	N/A	N/A
Linck/2023 [31]	1	65	L	3.2	RATH+BSO+OMT+P	IIB	-	Alive	N/A
Nagase/2023 [32]	1	48	R	20	RSO+OMT+P+colostomy (post TH+LSO)	IVB	-	Dead	15
The present study	3	52–76	L (1); R (1); B (1)	13, 15.5, 16.5	TH+BSO+OMT+PLNb (2); TH+BSO+OMT+PLNb+LAR (1)	IC1 (2); ypIIB (1)	Yes (2: liver, 2; lung, 1), No (1)	Alive (2); Dead (1)	7–44

Abbreviations: N/A, not available; B, bilateral; L, left; R, right; TH, total hysterectomy; BSO, bilateral salpingo-oophorectomy; OMT, omentectomy; P, peritoneal resection; PLND pelvic lymph node dissection; PLNb, pelvic lymph node biopsy; PALND, para-aortic lymph node dissection; LSO, left salpingo-oophorectomy.

**Table 5:** Genetic analysis and therapeutic targets in previously reported cases of mesonephric-like adenocarcinoma of the ovary.

Author/Year	*n*	HRD	MMR	PD-L1	HER2	ER	PR	Genetic Analysis	*KRAS* Mutation	Other Gene Alterations
McFarland/2016 [1]; Mirkovic/2018 [7]	5	N/A	N/A	N/A	N/A	Neg (4/4)	Neg (4/4)	TS (4); N/A (1)	4/4	*PIK3CA*
Pors/2018 [8]	1	N/A	N/A	N/A	N/A	Neg	N/A	N/A	N/A	N/A
Chapel/2018 [9]	1	N/A	N/A	N/A	N/A	Neg	Neg	TS	-	*NRAS, BCOR*
McCluggage/2020 [10]	5	N/A	N/A	N/A	N/A	Neg	Neg	TS (1); N/A (4)	1/1	*-*
Dundr/2020 [11]	1	N/A	N/A	N/A	N/A	Neg	Neg	TS	1/1	*PIK3CA, CHEK2*
Seay/2020 [12]	1	N/A	N/A	score 0	N/A	Neg	Neg	TS	-	*-*
Chen/2020 [13]	1	N/A	N/A	N/A	N/A	Neg	Neg	N/A	N/A	N/A
Qazi/2020 [14]	1	N/A	N/A	N/A	N/A	Neg (1/2); N/A (1/2)	Neg (1/2); N/A (1/2)	N/A	N/A	N/A
Pors/2021 [5]	25	N/A	N/A	N/A	N/A	N/A	N/A	N/A	N/A	N/A
da Silva/2021 [15]	15	N/A	Intact (8); N/A (7)	N/A	N/A	Focal (2/15); Neg (13/15)	Neg (12/15); N/A (3/15)	TS	13/15	*PIK3CA*, *SPOP*, *NRAS*, *SETD8*, *CTNNB1*, *CREBBP*, *NOTCH3*, *ARID1A*, *FBXW7*, *FANCA*, *AKT1*, *ASXL1*, *RAD54L*
Kim/2021 [16]	1	N/A	Intact	N/A	N/A	Neg	Neg	TS	1/1	*-*
Karpathiou/2021 [17]	1	HRP	Intact	N/A	N/A	Neg	Neg	TS	1/1	*CTNNB1*
Ujita/2021 [18]	1	N/A	N/A	N/A	N/A	Neg	Neg	N/A	N/A	N/A
Deolet/2022 [19]	4	N/A	N/A	N/A	N/A	Neg	Neg (3/4) N/A (1/4)	TS	3/4	*PIK3CA*, *PTEN amplification, 12p isochromosome*
Koh/2022 [20]	5	N/A	Intact (4); N/A (1)	N/A	N/A	Focal (3/5); Neg (2/5)	Focal (1/5); Neg (4/5)	TS (4); N/A (1)	4/4	*-*
Ishida/2022 [21]	1	N/A	N/A	N/A	N/A	Neg	Neg	TS	1/1	*SPOP, FANCA*
Arslanian/2022 [22]	2	N/A	Intact (2)	N/A	N/A	Focal (1/2); Neg (1/2)	Neg (1/2); N/A (1/2)	TS	2/2	*PIK3CA*
Nilforoushan/2022 [23]	2	N/A	N/A	N/A	N/A	Neg (2/2)	Neg (2/2)	TS (1); N/A (1)	1/2; N/A (1)	*CTNNB1*, *FGFR2 amplification*, *CDKN2A/ p16 deletion*
Mirkovic/2023 [24]	2	N/A	Intact	N/A	N/A	Neg (2/2)	Neg (1/2); N/A (1/2)	TS	2/2	*FANCA(1/2)*, *CREBBP(2/2)*, *POLE(1/2)*, *PTEN(1/2)*
Xu/2023 [25]	1	N/A	Intact	N/A	N/A	Neg	N/A	TS	-	*FGFR2*, *CTNNB1*
Nilforoushan/2023 [26]	1	N/A	Intact	Neg	N/A	Neg	Neg	TS	1/1	*NOTCH1*
Stolnicu/2023 [27]	1	N/A	N/A	N/A	N/A	Neg	Neg	TS	1/1	*RRR2R1A*, *ARHGAP35*, *IRS1*
Kommoss/2023 [28]	14	N/A	Intact (14)	N/A	N/A	N/A	N/A	N/A	N/A	N/A
Zhao/2023 [29]	1	N/A	Intact	N/A	N/A	Neg	Neg	N/A	N/A	N/A
Chang/2023 [30]	2	N/A	N/A	N/A	N/A	Neg	Neg (1/2); N/A (1/2)	TS (1); N/A (1)	1/1	*TP53*, *PPP2R1A*, *SPEN*
Linck/2023 [31]	1	N/A	N/A	N/A	N/A	Neg	Neg	N/A	N/A	N/A
Nagase/2023 [32]	1	N/A	N/A	N/A	N/A	Neg	Neg	TS	1/1	*PIK3CA*, *FBXW7*, *RAD21*
The present case	3	HRP (3)	Intact (3)	CPS < 1 (3)	Score 1+ (3)	Rare (1/3); Neg (2/3)	Neg (3/3)	N/A	N/A	N/A

Abbreviations: HRD, homologous recombination repair deficiency; MMR, mismatch repair; PD-L1, Programmed cell Death ligand 1; HER2, human epidermal growth factor receptor 2; TS, target sequencing; HRP, homologous recombination repair proficient.

## Data Availability

The data that support the findings of this study are available from the corresponding author upon reasonable request.

## Supplementary Materials

The following supporting information can be downloaded at: https://www.mdpi.com/article/10.3390/curroncol31090378/s1, Table S1: Morphological and immunohistochemical features of mesonephric-like adenocarcinoma; Table S2: Primary antibodies used for immunohistochemistry in this study.
