# Supplementary material for: Ovarian Mesonephric-like Adenocarcinoma: Its Prevalence in a Japanese High-Volume Cancer Center and a Literature Review on Therapeutic Targets
Source: Curr Oncol. 2024 Aug 30;31(9):5107–20. doi: 10.3390/curroncol31090378 (PMC11430596; doi:10.3390/curroncol31090378)
Supplement: Supplementary file 1 [file curroncol-31-00378-s001.zip › curroncol-3176918-Supplementary.pdf]

Supplementary Table S1. Morphological and immunohistochemical features of mesonephric-like adenocarcinoma

**Morphological features**
• Considerable morphologic similarity to cervical mesonephric adenocarcinoma is observed.<br>• Admixture of different architectural patterns are seen: Tubular, glandular (pseudoendometrioid), papillary, cribriform, slit-like, retiform, glomeruloid and solid, in various combinations<br>• Intraluminal eosinophilic colloid-like material is often identified.<br>• Squamous, ciliated and mucinous differentiation are generally absent.
**Cytological features**
• Cuboidal or columnar cells are frequently observed.<br>• Mild or moderately atypical angulated clear vesicular nuclei which often exhibit a degree of overlapping. Sometimes nuclear grooves are present.<br>• Hobnail cells may be present.<br>• The cytoplasm is usually scant to moderate and mitotic activity is usually conspicuous.<br>• Sometimes a component of cells with ovoid to spindle shaped nuclei is present, especially in the solid areas.
**Immunohistochemical features**
• Diffuse positive: PAX8 and CK7<br>• Diffuse or focal positive: TTF1, GATA3, CD10 (apical/luminal staining), calretinin<br>• Negative: ER, PR, CK20, WT-1<br>• Wild-type pattern of p53

Adapted from McCluggage WG. Mesonephric-like Adenocarcinoma of the Female Genital Tract: From Morphologic Observations to a Well-characterized Carcinoma With Aggressive Clinical Behavior. Adv Anat Pathol. 2022 Jul 1;29(4):208-216.

Supplementary table S2. Primary antibodies used for immunohistochemistry in this study

Marker	Clone	Source	Dilution	Antigen retrieval
PAX8	PAX8R1	Abcam, Cambridge, MA, USA	1:50	Tris-EDTA (pH 9)
ER	SP1	*Ventana* Medical Systems, Tucson, AZ, USA	Prediluted	Tris-EDTA (pH 9)
PR	1.00E+02	*Ventana* Medical Systems, Tucson, AZ, USA	Prediluted	Tris-EDTA (pH 9)
CD10	56C6	Dako, Glostrup, Denmark	Prediluted	Tris-EDTA (pH 9)
GATA3	HG3-31	Santa Cruz Biotechnology, Dallas, TX, USA	1:50	Citrate buffer (pH 6)
TTF-1	8G7G3/1	Dako, Glostrup, Denmark	Prediluted	Tris-EDTA (pH 9)
CDX2	DAK-CDX2	Dako, Glostrup, Denmark	Prediluted	Tris-EDTA (pH 9)
WT-1	6F-H2	Dako, Glostrup, Denmark	Prediluted	Tris-EDTA (pH 9)
p53	DO7	Dako, Glostrup, Denmark	Prediluted	Tris-EDTA (pH 9)
Ki-67	MIB-1	Dako, Glostrup, Denmark	Prediluted	Citrate buffer (pH 6)
P40	BC28	Abcam, Cambridge, MA, USA	1:200	Tris-EDTA (pH 9)
HER2	Hercep test	Dako, Glostrup, Denmark	Prediluted	Tris-EDTA (pH 9)
PMS2	EP51	Dako, Glostrup, Denmark	Prediluted	Tris-EDTA (pH 9)
MSH6	EP49	Dako, Glostrup, Denmark	Prediluted	Tris-EDTA (pH 9)
PD-L1	22C3	Dako, Glostrup, Denmark	Prediluted	Tris-EDTA (pH 9)
